# Research interest on dental sealant in dentistry based on the 100 most cited articles: bibliometric analysis

**DOI:** 10.1590/1807-3107bor-2024.vol38.0044

**Published:** 2024-05-13

**Authors:** Melissa Santos da Silva SIMÕES, Aurélio de Oliveira ROCHA, Mariana Perini ZENDRON, Pablo Silveira SANTOS, Michele BOLAN, Carla Miranda SANTANA, Mariane CARDOSO

**Affiliations:** (a) Universidade Federal de Santa Catarina – UFSC, Postgraduate Program in Dentistry, Florianopolis, SC, Brazil.; (b) Universidade Federal de Santa Catarina – UFSC, Department of Dentistry, Florianopolis, SC, Brazil.

**Keywords:** Pit and Fissure Sealants, Dental Caries, Dental Materials, Bibliometrics

## Abstract

The aim of this study was to identify and analyze the characteristics of the 100 most cited articles about dental sealants (DS) in dentistry. In September 2023, a search was performed in the Web of Science Core Collection (WoS-CC) database. The following information was extracted from each article: number and density of citations, year of publication, authorship, journal, impact factor, keywords, study design, theme, continent, country, and institution. The citations of the WoS-CC were compared with those of the Scopus and Google Scholar databases. The VOSviewer software was used to generate collaborative networks. The number of citations ranged from 33 to 205. The articles were published between 1961 and 2016. Buonocore MG (7%) was the most prominent author among the most cited. The Journal of the American Dental Association was the most frequent journal (25%) and Journal of Dental Research (7.6) had the highest impact factor. Most studies had interventional (41%) and laboratory (31%) designs, mainly addressing DS effectiveness in the prevention and control of dental caries (86%). There was a predominance of publications from North America (46%) and the USA was the country with the highest number of articles (44%). The most frequent institutions were the Centers for Disease Control and Prevention (USA) and the University of Rochester (USA) (6% each). “Retention” was the most frequent keyword. In conclusion, the 100 most cited articles were mostly interventional and laboratory studies, addressing the retention and efficacy of DS. Most of the articles were concentrated in North America and Europe, demonstrating a little collaboration from other continents.

## Introduction

A comprehensive review of the global epidemiology of dental caries showed a high prevalence of this condition, with untreated caries being the most common.^1^ According to Kassebaum et al.^2^ cavitated carious lesions in permanent teeth remained the most prevalent health condition worldwide in 2010, affecting 2.4 billion people. In addition, untreated cavitated carious lesion in dentin of primary teeth was the 10th most prevalent health condition, affecting 621 million children worldwide.^2^ Also, the tooth surface most affected by the disease was the occlusal surface of first permanent molars, which, due to their location, are less visible, making cleaning more difficult and increasing the risk of developing carious lesions.^3^

The use of dental sealants (DS) is an alternative for preventing and controlling initial carious lesions.^4^ Sealants, which are mainly based on resin or glass ionomer, were developed to be applied to the occlusal surfaces of teeth at risk of developing carious lesions, preventing plaque stagnation in these areas.^5^ The presence of pits and fissures on the occlusal surfaces of posterior teeth makes this area highly susceptible to biofilm accumulation and challenging to clean.^6^ In addition, posterior teeth are the most difficult to access, especially during eruption.^7^ DS act as a physical barrier, forming a protective layer that prevents food retention and bacterial proliferation in areas vulnerable to dental caries.^8^ DS are also an efficient alternative to paralyze non-cavitated carious lesions before destroying dental structures. ^9,10^

Systematic reviews agree on the recommendations of DS to prevent or control the development of carious lesions, but diverse clinical data are available on the application of DS and types of material used.^11,12^ Thus, due to the relatively large number of published studies on DS and specific information, mapping the scientific evidence through a bibliometric analysis is essential to identify trends in research and the existence of knowledge gaps on the subject, as already reported in other areas of dentistry.^13,14,15^ Such a study can help researchers identify the development and scientific status of publications on DS to stimulate new investigations. Thus, the present study aimed to analyze the 100 most cited articles in the literature on DS through a bibliometric analysis.

## Methodology

The primary search was conducted in September 2023. The database selected was the Clarivate Analytics Web of Science Core Collection (WoS-CC). The filter “Dentistry, oral surgery and medicine” was used. No restrictions were applied regarding language of studies and year of publication. The following search strategy was used: TS= (“Pit and Fissure Sealants” OR “Fissure Sealant” OR “Dental Sealant” OR “Teeth Sealants” OR “Dental Sealants” OR “Pit Fissure Sealants” OR “Sealant, Pit Fissure” OR “Fissure Sealant, Pit” OR “Sealants, Tooth” OR “Sealants, Dental” OR “Glass Ionomer Sealant” OR “Resin Sealant” OR “Sealing of Pits and Fissures” OR “Pit and Fissure Sealing” OR “” OR “” OR “Adhesive Sealing of Pits and Fissures” OR “

The list of articles obtained in WoS-CC was organized in descending order according to the number of citations. Articles addressing topics related to dental sealants were included. Editorial and conference articles were excluded. Two independent reviewers (MSSS and AOR) selected the 100 most cited articles. A third reviewer (MC) was consulted for the resolution of doubts. For the selection of the articles, the title and abstract were initially read, and the full texts were accessed when there were doubts about the eligibility criteria. Then, the full texts were read to collect the study data. In case of a tie in the number of citations, the position of the article in the list was based on the highest citation density of the WoS-CC (average number of citations received per year). Subsequently, a manual search was conducted on the same day on the number of citations of the selected articles in Scopus and Google Scholar databases to compare with WoS-CC and evaluate the order of citations of these articles in the other databases.^13-15^

The following items were extracted from each study: title, authors, number of authors, number of citations, citation density, institution, country, and continent (based on the corresponding author’s affiliation), year of publication, journal title, journal impact factor (2022), keywords, study design, and theme. The study designs were classified as follows: systematic reviews, literature reviews, laboratory studies, observational studies, intervention studies, and case reports. All articles were grouped according to the most prevalent themes: sealant retention and effectiveness against caries, the release of bisphenol-A, sealant application technique, and others. Topics that appeared only once were classified as “other”.

The Visualization of Similarities Viewer (VOSviewer) software was used to generate a graphical representation of collaborative networks between keywords and between authors (both with at least three occurrences). The most important terms for the keyword map were represented by larger circles, and the strongly related terms were interconnected and represented by similar coloring. Words in the larger circles were those with higher occurrence. In contrast, words in smaller circles presented a lower occurrence. Words connected by bibliometric networks indicated the use of both keywords in the same study. For the author’s map, names in the same cluster indicated collaboration between authors, and the author’s relevance was indicated by color; the closer to red, the more frequent this author was, and the closer to yellow the lower the frequency of this author in the 100 most cited articles.^16,17^

Statistical analysis was performed by comparing the number of citations among the three databases. The statistical software SPSS for Windows (SPSS, version 21.0; IBM Corp, Armonk, N.Y., USA) was used. The Kolmogorov-Smirnov test was used to verify the data distribution normality. Spearman’s correlation coefficient was used, as the data had non-normal distribution, and classified as very high positive correlation (between 0.900 to 1.000); high positive correlation (0.700 to 0.900); moderate positive correlation (0.500 to 0.700); low positive correlation (0.300 to 0.500); and negligible correlation (0.000 to 0.300).^18^ Significance level was set at 5%.

## Results

A total of 1,111 documents were retrieved and listed in descending order of number of citations. Subsequently, the 100 most cited articles on DS were selected (Table 1). The 100 articles were cited 6,513 times in the WoS-CC, including 384 self-citations (5.9%). The number of citations of the 100 most cited articles in WoS-CC ranged from 33 to 205, with 13% of articles cited at least 100 times. In the other databases, a greater number of citations were observed, 7,309 (ranging from 29 to 269) in Scopus and 15,610 (ranging from 48 to 526) in Google Scholar. There was a high positive correlation between number of citations in WoS-CC and Google Scholar (r=0.859) and between Google Scholar and Scopus (r = 0.890), and a very high positive correlation between WoS-CC and Scopus (r = 0.910).

**Table 1:** Top 100 most cited articles on Dental Sealant.

Position	Article	Number of citations (Citation density)		

		WoS Core Collection	Scopus	Google Scholar
1	Simonsen RJ. Retention and effectiveness of dental sealant after 15 years. J Am Dent Assoc. 1991 Oct;122(10):34-42.	205	269	526
		(6.41)	(8.68)	(16.44)
2	Griffin SO, Oong E, Kohn W, Vidakovic B, Gooch BF; CDC Dental Sealant Systematic Review Work Group, Bader J, Clarkson J, Fontana MR, Meyer DM, Rozier RG, Weintraub JA, Zero DT. The effectiveness of sealants in managing caries lesions. J Dent Res. 2008 Feb;87(2):169-74.	191	238	465
		(12.73)	(17.00)	(31.00)
3	Cueto EI, Buonocore MG. Sealing of pits and fissures with an adhesive resin: its use in caries prevention. J Am Dent Assoc. 1967 Jul;75(1):121-8.	172	160	424
		(3.07)	(2.91)	(7.57)
4	Ripa LW. Sealants revisted: an update of the effectiveness of pit-and-fissure sealants. Caries Res. 1993;27 Suppl 1:77-82.	151	186	391
		(5.03)	(6.41)	(13.03)
5	Joskow R, Barr DB, Barr JR, Calafat AM, Needham LL, Rubin C. Exposure to bisphenol A from bis-glycidyl dimethacrylate-based dental sealants. J Am Dent Assoc. 2006 Mar;137(3):353-62.	150	157	244
		(8.82)	(9.81)	(14.35)
6	Buonocore M. Adhesive sealing of pits and fissures for caries prevention, with use of ultraviolet light. J Am Dent Assoc. 1970 Feb;80(2):324-30.	146	111	319
		(2.75)	(2.13)	(6.02)
7	Buonocore MG. Caries prevention in pits and fissures sealed with an adhesive resin polymerized by ultraviolet light: a two-year study of a single adhesive application. J Am Dent Assoc. 1971 May;82(5):1090-3.	139	107	242
		(2.67)	(2.10)	(4.65)
8	Wright JT, Crall JJ, Fontana M, Gillette EJ, Nový BB, Dhar V, Donly K, Hewlett ER, Quinonez RB, Chaffin J, Crespin M, Iafolla T, Siegal MD, Tampi MP, Graham L, Estrich C, Carrasco-Labra A. Evidence-based clinical practice guideline for the use of pit-and-fissure sealants: A report of the American Dental Association and the American Academy of Pediatric Dentistry. J Am Dent Assoc. 2016 Aug;147(8):672-682.e12.	117	150	313
		(16.71)	(25.00)	(44.71)
9	Paris S, Meyer-Lueckel H. Inhibition of caries progression by resin infiltration in situ. Caries Res. 2010;44(1):47-54.	115	132	276
		(8.85)	(11.00)	(21.23)
10	Mejàre I, Lingström P, Petersson LG, Holm AK, Twetman S, Källestål C, Nordenram G, Lagerlöf F, Söder B, Norlund A, Axelsson S, Dahlgren H. Caries-preventive effect of fissure sealants: a systematic review. Acta Odontol Scand. 2003 Dec;61(6):321-30.	109	134	319
		(5.45)	(7.05)	(15.95)
11	Oong EM, Griffin SO, Kohn WG, Gooch BF, Caufield PW. The effect of dental sealants on bacteria levels in caries lesions: a review of the evidence. J Am Dent Assoc. 2008 Mar;139(3):271-8; quiz 357-8.	108	143	309
		(7.20)	(10.21)	(20.60)
12	Gooch BF, Griffin SO, Gray SK, Kohn WG, Rozier RG, Siegal M, Fontana M, Brunson D, Carter N, Curtis DK, Donly KJ, Haering H, Hill LF, Hinson HP, Kumar J, Lampiris L, Mallatt M, Meyer DM, Miller WR, Sanzi-Schaedel SM, Simonsen R, Truman BI, Zero DT; Centers for Disease Control and Prevention. Preventing dental caries through school-based sealant programs: updated recommendations and reviews of evidence. J Am Dent Assoc. 2009 Nov;140(11):1356-65.	108	133 (10.23)	264
		(7.71)		(18.86)
13	Fung EY, Ewoldsen NO, St Germain HA Jr, Marx DB, Miaw CL, Siew C, Chou HN, Gruninger SE, Meyer DM. Pharmacokinetics of bisphenol A released from a dental sealant. J Am Dent Assoc. 2000 Jan;131(1):51-8.	104	114	212
		(4.52)	(5.18)	(9.22)
14	Llodra JC, Bravo M, Delgado-Rodriguez M, Baca P, Galvez R. Factors influencing the effectiveness of sealants--a meta-analysis. Community Dent Oral Epidemiol. 1993 Oct;21(5):261-8.	96	121 (4.17)	263
		(3.20)		(8.77)
15	Paris S, Meyer-Lueckel H, Cölfen H, Kielbassa AM. Penetration coefficients of commercially available and experimental composites intended to infiltrate enamel carious lesions. Dent Mater. 2007 Jun;23(6):742-8.	95	105 (7.00)	228
		(5.94)		(14.25)
16	Going RE, Loesche WJ, Grainger DA, Syed SA. The viability of microorganisms in carious lesions five years after covering with a fissure sealant. J Am Dent Assoc. 1978;97(3):455-462.	94	102	252
		(2.09)	(2.32)	(5.60)
17	Waggoner WF, Siegal M. Pit and fissure sealant application: updating the technique. J Am Dent Assoc. 1996 Mar;127(3):351-61, quiz 391-2.	84	131 (5.04)	284
		(3.11)		(10.52)
18	Liu BY, Lo EC, Chu CH, Lin HC. Randomized trial on fluorides and sealants for fissure caries prevention. J Dent Res. 2012 Aug;91(8):753-8.	82	96	225
		(7.45)	(9.60)	(20.45)
19	Kühnisch J, Mansmann U, Heinrich-Weltzien R, Hickel R. Longevity of materials for pit and fissure sealing--results from a meta-analysis. Dent Mater. 2012 Mar;28(3):298-303.	81	100	197
		(7.36)	(10.00)	(17.91)
20	Cury JA, de Oliveira BH, dos Santos AP, Tenuta LM. Are fluoride releasing dental materials clinically effective on caries control? Dent Mater. 2016 Mar;32(3):323-33.	81	96	169
		(11.57)	(16.00)	(24.14)
21	Kaga M, Kakuda S, Ida Y, Toshima H, Hashimoto M, Endo K, Sano H. Inhibition of enamel demineralization by buffering effect of S-PRG filler-containing dental sealant. Eur J Oral Sci. 2014 Feb;122(1):78-83.	80	81 (10.13)	124
		(8.89)		(13.78)
22	Nathanson D, Lertpitayakun P, Lamkin MS, Edalatpour M, Chou LL. In vitro elution of leachable components from dental sealants. J Am Dent Assoc. 1997 Nov;128(11):1517-23.	79	92 (3.68)	151
		(3.04)		(5.81)
23	Azarpazhooh A, Main PA. Pit and fissure sealants in the prevention of dental caries in children and adolescents: a systematic review. J Can Dent Assoc. 2008 Mar;74(2):171-7.	79	97 (6.93)	261
		(5.27)		(17.40)
24	Wright JT, Tampi MP, Graham L, Estrich C, Crall JJ, Fontana M, Gillette EJ, Nový BB, Dhar V, Donly K, Hewlett ER, Quinonez RB, Chaffin J, Crespin M, Iafolla T, Siegal MD, Carrasco-Labra A. Sealants for preventing and arresting pit-and-fissure occlusal caries in primary and permanent molars: A systematic review of randomized controlled trials-a report of the American Dental Association and the American Academy of Pediatric Dentistry. J Am Dent Assoc. 2016 Aug;147(8):631-645.e18.	79	53 (8.83)	267
		(11.29)		(38.14)
25	Meyer-Lueckel H, Paris S, Mueller J, Cölfen H, Kielbassa AM. Influence of the application time on the penetration of different dental adhesives and a fissure sealant into artificial subsurface lesions in bovine enamel. Dent Mater. 2006 Jan;22(1):22-8.	77	85 (5.31)	166 (9.76)
		(4.53)		
26	Shimazu K, Ogata K, Karibe H. Evaluation of the ion-releasing and recharging abilities of a resin-based fissure sealant containing S-PRG filler. Dent Mater J. 2011;30(6):923-7.	76	76 (6.91)	135
		(6.33)		(11.25)
27	Hamid A, Hume WR. A study of component release from resin pit and fissure sealants in vitro. Dent Mater. 1997 Mar;13(2):98-102.	75	81 (3.24)	162
		(2.88)		(6.23)
28	Kühnisch J, Berger S, Goddon I, Senkel H, Pitts N, Heinrich-Weltzien R. Occlusal caries detection in permanent molars according to WHO basic methods, ICDAS II and laser fluorescence measurements. Community Dent Oral Epidemiol. 2008 Dec;36(6):475-84.	73	88 (6.29)	179
		(4.87)		(11.93)
29	Weintraub JA. The effectiveness of pit and fissure sealants. J Public Health Dent. 1989;49(5 Spec No):317-30.	71	80 (2.42)	181
		(2.09)		(5.32)
30	Brown LJ, Kaste LM, Selwitz RH, Furman LJ. Dental caries and sealant usage in U.S. children, 1988-1991: selected findings from the Third National Health and Nutrition Examination Survey. J Am Dent Assoc. 1996 Mar;127(3):335-43.	71	24 (0.92)	146
		(2.63)		(5.41)
31	Forss H, Saarni UM, Seppä L. Comparison of glass-ionomer and resin-based fissure sealants: a 2-year clinical trial. Community Dent Oral Epidemiol. 1994 Feb;22(1):21-4.	70	79 (2.82)	156
		(2.41)		(5.38)
32	Mertz-Fairhurst EJ, Fairhurst CW, Williams JE, Della-Giustina VE, Brooks JD. A comparative clinical study of two pit and fissure sealants: 7-year results in Augusta, GA. J Am Dent Assoc. 1984 Aug;109(2):252-5.	68	72 (1.89)	133
		(1.74)		(3.41)
33	Splieth CH, Ekstrand KR, Alkilzy M, Clarkson J, Meyer-Lueckel H, Martignon S, Paris S, Pitts NB, Ricketts DN, van Loveren C. Sealants in dentistry: outcomes of the ORCA Saturday Afternoon Symposium 2007. Caries Res. 2010;44(1):3-13.	68	76 (6.33)	150
		(5.23)		(11.54)
34	Bravo M, Montero J, Bravo JJ, Baca P, Llodra JC. Sealant and fluoride varnish in caries: a randomized trial. J Dent Res. 2005 Dec;84(12):1138-43.	64	69	136
		(3.56)	(4.06)	(7.56)
35	Poulsen S, Beiruti N, Sadat N. A comparison of retention and the effect on caries of fissure sealing with a glass-ionomer and a resin-based sealant. Community Dent Oral Epidemiol. 2001;29(4):298-301.	63	82	192
		(2.86)	(3.90)	(8.73)
36	Atkinson JC, Diamond F, Eichmiller F, Selwitz R, Jones G. Stability of bisphenol A, triethylene-glycol dimethacrylate, and bisphenol A dimethacrylate in whole saliva. Dent Mater. 2002 Mar;18(2):128-35.	63	64 (3.20)	110
		(3.00)		(5.24)
37	Antonson SA, Antonson DE, Brener S, Crutchfield J, Larumbe J, Michaud C, Yazici AR, Hardigan PC, Alempour S, Evans D, Ocanto R. Twenty-four month clinical evaluation of fissure sealants on partially erupted permanent first molars: glass ionomer versus resin-based sealant. J Am Dent Assoc. 2012 Feb;143(2):115-22.	62	72 (7.20)	118
		(5.64)		(10.73)
38	Muller-Bolla M, Lupi-Pégurier L, Tardieu C, Velly AM, Antomarchi C. Retention of resin-based pit and fissure sealants: A systematic review. Community Dent Oral Epidemiol. 2006 Oct;34(5):321-36.	61	68 (4.25)	176
		(3.59)		(10.35)
39	Paris S, Meyer-Lueckel H, Mueller J, Hummel M, Kielbassa AM. Progression of sealed initial bovine enamel lesions under demineralizing conditions in vitro. Caries Res. 2006;40(2):124-9.	61	69 (4.31)	177
		(3.59)		(10.41)
40	Arrow P, Riordan PJ. Retention and caries preventive effects of a GIC and a resin-based fissure sealant. Community Dent Oral Epidemiol. 1995 Oct;23(5):282-5.	60	78 (2.89)	142
		(2.14)		(5.07)
41	Mueller J, Meyer-Lueckel H, Paris S, Hopfenmuller W, Kielbassa AM. Inhibition of lesion progression by the penetration of resins in vitro: influence of the application procedure. Oper Dent. 2006;31(3):338-345.	60	70	176
		(3.53)	(4.38)	(10.35)
42	Heller KE, Reed SG, Bruner FW, Eklund SA, Burt BA. Longitudinal evaluation of sealing molars with and without incipient dental caries in a public health program. J Public Health Dent. 1995 Summer;55(3):148-53.	59	81 (3.00)	164
		(2.11)		(5.86)
43	Zhou SL, Zhou J, Watanabe S, Watanabe K, Wen LY, Xuan K. In vitro study of the effects of fluoride-releasing dental materials on remineralization in an enamel erosion model. J Dent. 2012 Mar;40(3):255-63.	59	68 (6.80)	140
		(5.36)		(12.73)
44	Chow LC, Brown WE. Phosphoric acid conditioning of teeth for pit and fissure sealants. J Dent Res. 1973 Sep-Oct;52(5):1158.	58	55 (1.12)	154
		(1.16)		(3.08)
45	Brown LJ, Selwitz RH. The impact of recent changes in the epidemiology of dental caries on guidelines for the use of dental sealants. J Public Health Dent. 1995;55(5 Spec No):274-91.	57	69 (2.56)	147
		(2.04)		(5.25)
46	Tarumi H, Imazato S, Narimatsu M, Matsuo M, Ebisu S. Estrogenicity of fissure sealants and adhesive resins determined by reporter gene assay. J Dent Res. 2000 Nov;79(11):1838-43.	57	60 (2.73)	106
		(2.48)		(4.61)
47	Mertz-Fairhurst EJ, Adair SM, Sams DR, Curtis JW Jr, Ergle JW, Hawkins KI, Mackert JR, O’Dell NL, Richards EE, Rueggeberg F, et al. Cariostatic and ultraconservative sealed restorations: nine-year results among children and adults. ASDC J Dent Child. 1995 Mar-Apr;62(2):97-107.	56	62 (2.30)	109
		(2.00)		(3.89)
48	Going RE, Haugh LD, Grainger DA, Conti AJ. Four-year clinical evaluation of a pit and fissure sealant. J Am Dent Assoc. 1977 Nov;95(5):972-81.	55	41 (0.91)	93
		(1.20)		(2.02)
49	Beiruti N, Frencken JE, van’t Hof MA, Taifour D, van Palenstein Helderman WH. Caries-preventive effect of a one-time application of composite resin and glass ionomer sealants after 5 years. Caries Res. 2006;40(1):52-9.	55	66 (4.13)	153
		(3.24)		(9.00)
50	Simonsen RJ, Neal RC. A review of the clinical application and performance of pit and fissure sealants. Aust Dent J. 2011;56 Suppl 1:45-58.	55	67	195
		(4.58)	(6.09)	(16.25)
51	Beiruti N, Frencken JE, van ‘t Hof MA, van Palenstein Helderman WH. Caries-preventive effect of resin-based and glass ionomer sealants over time: a systematic review. Community Dent Oral Epidemiol. 2006;34(6):403-409.	52	56 (3.50)	134
		(3.06)		(7.88)
52	Celiberti P, Lussi A. Use of a self-etching adhesive on previously etched intact enamel and its effect on sealant microleakage and tag formation. J Dent. 2005;33(2):163-171.	52	51 (3.00)	108
		(2.89)		(6.00)
53	Papacchini F, Goracci C, Sadek FT, Monticelli F, Garcia-Godoy F, Ferrari M. Microtensile bond strength to ground enamel by glass-ionomers, resin-modified glass-ionomers, and resin composites used as pit and fissure sealants. J Dent. 2005;33(6):459-467.	51	62 (3.76)	156
		(2.83)		(8.67)
54	Wendt LK, Koch G. Fissure sealant in permanent first molars after 10 years. Swed Dent J. 1988;12(5):181-185.	51	62 (1.82)	92
		(1.46)		(2.63)
55	Wendt LK, Koch G, Birkhed D. On the retention and effectiveness of fissure sealant in permanent molars after 15-20 years: a cohort study. Community Dent Oral Epidemiol. 2001;29(4):302-307.	51	70 (3.33)	139
		(2.32)		(6.32)
56	Beun S, Bailly C, Devaux J, Leloup G. Physical, mechanical and rheological characterization of resin-based pit and fissure sealants compared to flowable resin composites. Dent Mater. 2012;28(4):349-359.	51	58 (5.80)	111
		(4.64)		(10.09)
57	Yang SY, Piao YZ, Kim SM, Lee YK, Kim KN, Kim KM. Acid neutralizing, mechanical and physical properties of pit and fissure sealants containing melt-derived 45S5 bioactive glass. Dent Mater. 2013;29(12):1228-1235.	51	52 (5.78)	75
		(5.10)		(7.50)
58	Buren JL, Staley RN, Wefel J, Qian F. Inhibition of enamel demineralization by an enamel sealant, Pro Seal: an in-vitro study. Am J Orthod Dentofacial Orthop. 2008;133(4 Suppl):S88-S94.	50	57 (4.07)	140
		(3.33)		(9.33)
59	Beun S, Bailly C, Devaux J, Leloup G. Rheological properties of flowable resin composites and pit and fissure sealants. Dent Mater. 2008;24(4):548-555.	49	54 (3.86)	109
		(3.27)		(7.27)
60	Kloukos D, Pandis N, Eliades T. In vivo bisphenol-a release from dental pit and fissure sealants: a systematic review. J Dent. 2013;41(8):659-667.	49	55 (6.11)	93
		(4.90)		(9.30)
61	Songpaisan Y, Bratthall D, Phantumvanit P, Somridhivej Y. Effects of glass ionomer cement, resin-based pit and fissure sealant and HF applications on occlusal caries in a developing country field trial. Community Dent Oral Epidemiol. 1995;23(1):25-29.	48	63 (2.33)	120
		(1.71)		(4.29)
62	Handelman SL, Buonocore MG, Heseck DJ. A preliminary report on the effect of fissure sealant on bacteria in dental caries. J Prosthet Dent. 1972;27(4):390-392.	48	35 (0.70)	107
		(0.94)		(2.10)
63	Bakhshandeh A, Qvist V, Ekstrand KR. Sealing occlusal caries lesions in adults referred for restorative treatment: 2-3 years of follow-up. Clin Oral Investig. 2012;16(2):521-529.	48	68 (6.80)	128
		(4.36)		(11.64)
64	Williams B, Laxton L, Holt RD, Winter GB. Fissure sealants: a 4-year clinical trial comparing an experimental glass polyalkenoate cement with a bis glycidyl methacrylate resin used as fissure sealants. Br Dent J. 1996;180(3):104-108.	46	59 (2.27)	105
		(1.70)		(3.89)
65	Forss H, Halme E. Retention of a glass ionomer cement and a resin-based fissure sealant and effect on carious outcome after 7 years. Community Dent Oral Epidemiol. 1998;26(1):21-25.	46	63 (2.63)	142
		(1.84)		(5.68)
66	Tulunoğlu O, Bodur H, Uçtaşli M, Alaçam A. The effect of bonding agents on the microleakage and bond strength of sealant in primary teeth. J Oral Rehabil. 1999;26(5):436-441.	45	63 (2.74)	112
		(1.88)		(4.67)
67	Frazier MC, Southard TE, Doster PM. Prevention of enamel demineralization during orthodontic treatment: an in vitro study using pit and fissure sealants. Am J Orthod Dentofacial Orthop. 1996;110(5):459-465.	45	47 (1.81)	134
		(1.67)		(4.96)
68	Locker D, Jokovic A, Kay EJ. Prevention. Part 8: The use of pit and fissure sealants in preventing caries in the permanent dentition of children. Br Dent J. 2003;195(7):375-378.	45	57 (3.00)	203
		(2.25)		(10.15)
69	Weerheijm KL, de Soet JJ, van Amerongen WE, de Graaff J. Sealing of occlusal hidden caries lesions: an alternative for curative treatment?. ASDC J Dent Child. 1992;59(4):263-268.	44	60 (2.00)	95
		(1.42)		(3.06)
70	Primosch RE, Barr ES. Sealant use and placement techniques among pediatric dentists. J Am Dent Assoc. 2001;132(10):1442-1461.	44	43 (2.05)	79
		(2.00)		(3.59)
71	Bravo M, Baca P, Llodra JC, Osorio E. A 24-month study comparing sealant and fluoride varnish in caries reduction on different permanent first molar surfaces. J Public Health Dent. 1997;57(3):184-186.	44	52 (2.08)	128
		(1.69)		(4.92)
72	Griffin SO, Gray SK, Malvitz DM, Gooch BF. Caries risk in formerly sealed teeth. J Am Dent Assoc. 2009;140(4):415-423.	44	58 (4.46)	118
		(3.14)		(8.43)
73	Geiger SB, Gulayev S, Weiss EI. Improving fissure sealant quality: mechanical preparation and filling level. J Dent. 2000;28(6):407-412.	43	56 (2.55)	129
		(1.87)		(5.61)
74	Handleman SL, Buonocore MG, Schoute PC. Progress report on the effect of a fissure sealant on bacteria in dental caries. J Am Dent Assoc. 1973;87(6):1189-1191.	43	29 (0.59)	95
		(0.86)		(1.90)
75	Benham AW, Campbell PM, Buschang PH. Effectiveness of pit and fissure sealants in reducing white spot lesions during orthodontic treatment. A pilot study. Angle Orthod. 2009;79(2):338-345.	43	44 (3.38)	127
		(3.07)		(9.07)
76	Karlzén-Reuterving G, van Dijken JW. A three-year follow-up of glass ionomer cement and resin fissure sealants. ASDC J Dent Child. 1995;62(2):108-110.	42	57 (2.11)	112
		(1.50)		(4.00)
77	Fontana M, Platt JA, Eckert GJ, et al. Monitoring of sound and carious surfaces under sealants over 44 months. J Dent Res. 2014;93(11):1070-1075.	42	58 (7.25)	72
		(4.67)		(8.00)
78	Messer LB, Calache H, Morgan MV. The retention of pit and fissure sealants placed in primary school children by Dental Health Services, Victoria. Aust Dent J. 1997;42(4):233-239.	41	56 (2.24)	97
		(1.58)		(3.73)
79	Hallström U. Adverse reaction to a fissure sealant: report of case. ASDC J Dent Child. 1993;60(2):143-146.	41	43 (1.48)	90
		(1.37)		(3.00)
80	Chen X, Du M, Fan M, Mulder J, Huysmans MC, Frencken JE. Effectiveness of two new types of sealants: retention after 2 years. Clin Oral Investig. 2012;16(5):1443-1450.	40	47 (4.70)	72
		(3.64)		(6.55)
81	Frencken JE, Wolke J. Clinical and SEM assessment of ART high-viscosity glass-ionomer sealants after 8-13 years in 4 teeth. J Dent. 2010;38(1):59-64.	39	47 (3.92)	93
		(3.00)		(7.15)
82	Hannig M, Gräfe A, Atalay S, Bott B. Microleakage and SEM evaluation of fissure sealants placed by use of self-etching priming agents. J Dent. 2004;32(1):75-81.	39	49	130
		(2.05)	(2.72)	(6.84)
83	Griffin SO, Griffin PM, Gooch BF, Barker LK. Comparing the costs of three sealant delivery strategies. J Dent Res. 2002;81(9):641-645.	39	42 (2.10)	93
		(1.86)		(4.43)
84	Simonsen RJ. The clinical effectiveness of a colored pit and fissure sealant at 36 months. J Am Dent Assoc. 1981;102(3):323-327.	38	34	66
		(0.90)	(0.83)	(1.57)
85	Peutzfeldt A, Nielsen LA. Bond strength of a sealant to primary and permanent enamel: phosphoric acid versus self-etching adhesive. Pediatr Dent. 2004;26(3):240-244.	38	45 (2.50)	99
		(2.00)		(5.21)
86	Komurcuoglu E, Olmez S, Vural N. Evaluation of residual monomer elimination methods in three different fissure sealants in vitro. J Oral Rehabil. 2005;32(2):116-121.	38	40 (2.35)	87
		(2.11)		(4.83)
87	Gwinnett AJ, Buonocore MG. A scanning electron microscope study of pit and fissure surfaces conditioned for adhesive sealing. Arch Oral Biol. 1972;17(3):415-423.	37	30	69
		(0.73)	(0.60)	(1.35)
88	Komatsu H, Shimokobe H, Kawakami S, Yoshimura M. Caries-preventive effect of glass ionomer sealant reapplication: study presents three-year results. J Am Dent Assoc. 1994;125(5):543-549.	37	43	97
		(1.28)	(1.54)	(3.34)
89	Bravo M, Garcia-Anllo I, Baca P, Llodra JC. A 48-month survival analysis comparing sealant (Delton) with fluoride varnish (Duraphat) in 6- to 8-year-old children. Community Dent Oral Epidemiol. 1997;25(3):247-250.	36	40 (1.60)	82
		(1.38)		(3.15)
90	McCune RJ, Bojanini J, Abodeely RA. Effectiveness of a pit and fissure sealant in the prevention of caries: three-year clinical results. J Am Dent Assoc. 1979;99(4):619-623.	36	33	59
		(0.82)	(0.77)	(1.34)
91	McCune RJ, Horowitz HS, Heifetz SB, Cvar J. Pit and fissure sealants: one-year results from a study in Kalispell, Montana. J Am Dent Assoc. 1973;87(6):1177-1180.	36	19	48
		(0.72)	(0.39)	(0.96)
92	Parkhouse RC, Winter GB. A fissure sealant containing methyl-2-cyanoacrylate as a caries preventive agent. A clinical evaluation. Br Dent J. 1971;130(1):16-19.	36	26 (0.51)	54 (1.04)
		(0.69)		
93	Bhuridej P, Kuthy RA, Flach SD, et al. Four-year cost-utility analyses of sealed and nonsealed first permanent molars in Iowa Medicaid-enrolled children. J Public Health Dent. 2007;67(4):191-198.	36	38 (2.53)	69
		(2.25)		(4.31)
94	Salar DV, García-Godoy F, Flaitz CM, Hicks MJ. Potential inhibition of demineralization in vitro by fluoride-releasing sealants. J Am Dent Assoc. 2007;138(4):502-506.	36	41 (2.73)	110
		(2.25)		(6.88)
95	Bravo M, Llodra JC, Baca P, Osorio E. Effectiveness of visible light fissure sealant (Delton) versus fluoride varnish (Duraphat): 24-month clinical trial. Community Dent Oral Epidemiol. 1996;24(1):42-46.	35	42 (1.62)	72
		(1.30)		(2.67)
96	Dewji HR, Drummond JL, Fadavi S, Punwani I. Bond strength of Bis-GMA and glass ionomer pit and fissure sealants using cyclic fatigue. Eur J Oral Sci. 1998;106(1):594-599.	35	36	49
		(1.40)	(1.50)	(1.96)
97	Flório FM, Pereira AC, Meneghim Mde C, Ramacciato JC. Evaluation of non-invasive treatment applied to occlusal surfaces. ASDC J Dent Child. 2001;68(5-6):326-301.	34	40	67
		(1.55)	(1.90)	(3.05)
98	Rock WP, Foulkes EE, Perry H, Smith AJ. A comparative study of fluoride-releasing composite resin and glass ionomer materials used as fissure sealants. J Dent. 1996;24(4):275-280.	34	49	80
		(1.26)	(1.88)	(2.96)
99	Selwitz RH, Winn DM, Kingman A, Zion GR. The prevalence of dental sealants in the US population: findings from NHANES III, 1988-1991. J Dent Res. 1996;75 Spec No:652-660.	33	39	62
		(1.22)	(1.50)	(2.30)
100	Gillings B, Buonocore M. Thickness of enamel at the base of pits and fissures in human molars and bicuspids. J Dent Res. 1961;40:119-133.	33	29	81
		(0.53)	(0.48)	(1.31)

**Figure 1:**
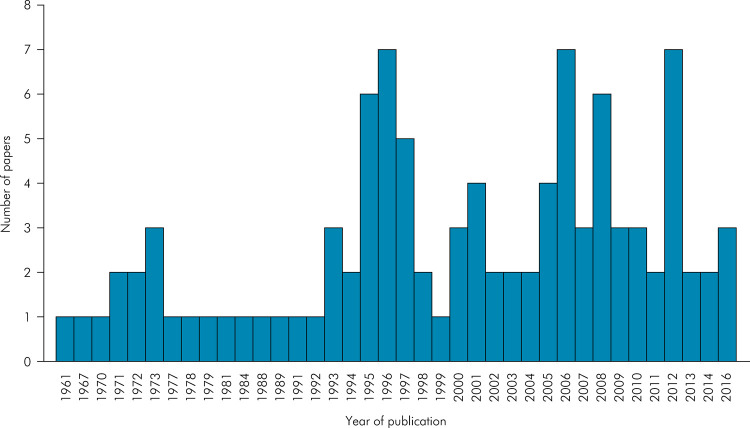
Distribution of the number of publications among the years.

The most cited article in WoS-CC (205 citations) was entitled “Retention and effectiveness of dental sealants after 15 years”, an intervention study by Simonsen RJ, published in 1991, which had an average of 6.61 citations per year, being also the most cited in Scopus (269 citations) and Google Scholar (526 citations). The earliest paper was published in 1961, “Thickness of enamel at the base of pits and fissures in human molars and bicuspids”, by Gillings B and Buonocore MG. Among the 100 most cited articles on DS, the highest number of publications was concentrated between 2001 to 2010 (36%), followed by 1991 to 2000 (31%) (Figure 1).

The Journal of the American Dental Association was the most prominent journal in several publications and citations in the top 100 (25%; 2,155 citations), followed by Community Dentistry and Oral Epidemiology (12%; 691 citations) (Table 2). According to the Journal of Citation Reports (2022), the journal with the highest impact factor in this study was the Journal of Dental Research (7.6), while the most frequent journal has an impact factor of 3.9.

**Table 2:** Top 10 journals with the highest number of articles in the list of the 100 most cited.

Journal name	Number of articles	Number of citations	Impact factor
Journal of the American Dental Association	25	2.155	3.9
Community Dentistry and Oral Epidemiology	12	691	2.3
Dental Materials	9	623	5.0
Journal of Dental Research	9	599	7.6
Journal of Dentistry	8	366	4.4
Caries Research	5	450	4.2
Journal of Public Health Dentistry	5	267	2.3
Journal of Dentistry for Children	5	217	0.8
British Dental Journal	3	127	2.6
European Journal of Oral Sciences	2	115	1.9

Most studies had an interventional design (42%; 2,693 citations), followed by laboratory studies (31%; 1,735 citations), literature reviews (13%; 990 citations), systematic reviews (9%; 825 citations), and observational studies (5%; 270 citations). Most of the studies addressed the theme retention and efficacy against caries (86%; 5,551 citations), followed by release of bisphenol-A (7%; 577 citations), dental sealant application technique (5%; 279 citations), and other themes (2%; 106 citations).

**Figure 2:**
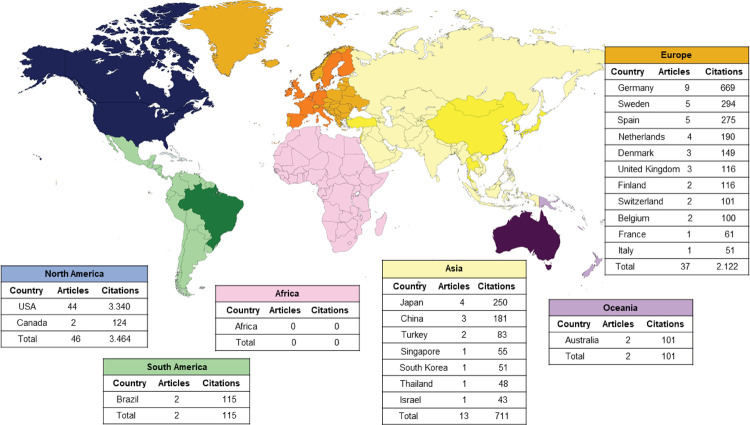
Map of countries and continents present among the top 100 most cited articles.

Among the continents with the most articles, Figure 2 shows that North America (44%) had the highest number of citations in WoS-CC (3,464 citations), Scopus (3,739 citations), and Google Scholar (8,324 citations), followed by Europe (37%) with second highest number of citations in WoS-CC (2,122 citations), Scopus (2,514 citations), and Google Scholar (5,296 citations). Considering the number of publications by country, the United States led with 44 articles (44%) that together add up to 3,340 citations in the WoS-CC, followed by Germany (9%; 669 citations). A total of 66 institutions were associated with the studies. Table 3 presents the ten institutions with the highest number of publications and citations. We highlight the Center for Disease Control and Prevention of the University of Rochester with six publications each (640 and 581 citations, respectively).

**Table 3:** Top 10 institutions with the highest number of articles among the 100 most cited.

Institution	Country	Number of articles	Number of citations
Centers for Disease Control and Prevention	USA	6	640
University of Rochester	USA	6	581
University Granada	Spain	5	275
University Med Berlin	Germany	4	293
Radboud University Nijmegen	Netherlands	3	146
University of Iowa	USA	3	131
University Munich	Germany	2	154
National Institute of Dental Research	USA	2	128
University Toronto	Canada	2	124
University Kuopio	Finland	2	116

**Figure 3:**
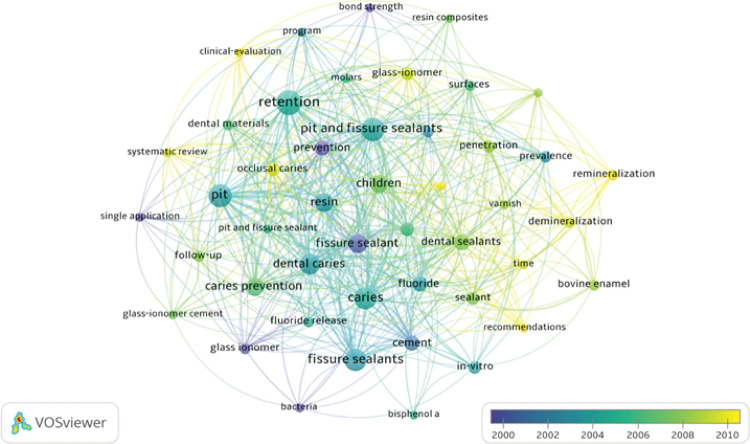
Density map of most used keywords among the 100 most cited articles.

Regarding keywords of the 100 most cited DS articles, 341 different terms appeared, with emphasis on “Retention” (25 occurrences), followed by “Pit” and “Pit and Fissure Sealants” (22 occurrences each). Figure 3, generated in VOSviewer, presents the most frequent keywords and their relationship. The words related by color represent the period of greatest occurrence for the terms. The dark blue color represents the period closer to the year 2000 and the yellow color, the period closer to the year 2010.

**Figure 4:**
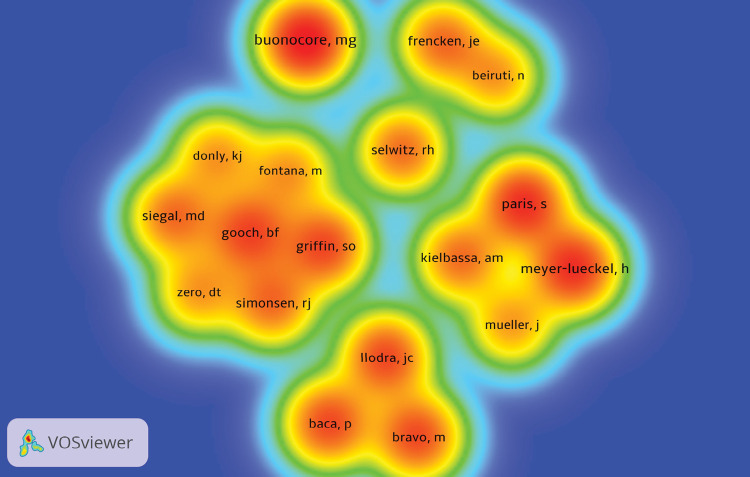
Density map of authors and collaborative co-authorships for the 100 most cited articles.

Three-hundred fifty-four authors were identified among the 100 most cited DS articles. Their frequency of appearance and the co-authorships between them is represented in Figure 4. The names represented by red/orange coloration are associated with authors with the highest occurrence, Buonocore MG, Meyer-Lueckel H, and Paris S. In contrast, the names associated with yellow correspond to authors with a lower occurrence. In addition, the interrelation between the groups of authors who collaborated when associated with the same cluster is shown. Table 4 shows the top 10 authors with the highest number of publications among the 100 most cited articles on DS. Buonocore MG, with seven articles, was the author with the highest number of publications, accounting for 618 citations, followed by Meyer-Lueckel H and Paris S, with six articles and 476 citations each.

**Table 4:** Top 10 authors with the highest number of articles among the 100 most cited.

Authors	Number of articles among the 100 most cited	Number of citations among the 100 most cited	Number of articles in WoS-CC	Number of citations in WoS-CC	H-Index
Buonocore, MG	7	618	33	1,146	21
Meyer-Lueckel, H	6	476	142	2,055	36
Paris, S	6	476	146	3,017	41
Gooch, BF	5	490	39	947	19
Griffin, SO	5	490	56	1,541	21
Baca, P	5	275	82	1,259	26
Bravo, M	5	275	127	2,665	31
Llodra, JC	5	275	35	848	18
Siegal, MD	4	388	128	4,495	28
Kielbassa, AM	4	293	184	3,78	42

## Discussion

This is the first bibliometric study to identify and discuss the 100 most cited articles related to DS through a quantitative approach. It can be stated that the high citation rates of these studies have been significantly associated with scientific development in the field of DS in dentistry, mostly addressing the clinical implication of the use of DS against dental caries. The total number of citations was higher in Google Scholar and Scopus when compared to WoS-CC. This can be justified by the fact that each database has exclusive and distinct methods for recording and counting citations.^13^ However, strong positive correlations were found between the number of citations in WoS-CC and the other two databases.

Articles that exceed 100 citations in a given area are considered classics.^19^ The most cited paper of this study stands out with 200 citations.^20^ Although published 31 years ago, this research had a 15-year follow-up with a sample of 200 patients.^20^ In another article,^21^ an extended follow-up between 15 and 20 years was also conducted, but with a sample of 70 patients and published ten years after the most cited article.^21^ The extended follow-up period may justify the large number of citations in the most cited article. Among the most cited articles, 35% had follow-up periods. However, most of these studies had follow-ups of one to three years. For many years, it has been discussed whether the use of DS is necessary and effective. Therefore, many studies have been conducted addressing this theme.^2,4-6^ Currently, WHO considers sealing the occlusal surface as a primary preventive measure, and one of the most effective and least invasive means available to ensure the protection of this surface against tooth decay.^22^

The oldest article was published by Gillings B and Buonocore M^23^ and mainly addressed the anatomy of the occlusal surface with its pits and fissures with the purpose of indicating an ideal material for the adequate filling of these structures during dental sealing. According to Liu BY et al.,^24^ the often deep and sinuous anatomy of pits and fissures is the most significant risk factor for dental caries, since controlling the accumulation and the removal of dental biofilm in these areas is often difficult or even unsatisfactory. To Beslot-Neveu A et al.,^25^ the DS must have a high degree of wettability and a degree of viscosity that allows its penetration into microcracks of the tooth enamel, ensuring a complete seal and strong adhesion of the material. In addition, the surface tension of the tooth structure must be reduced to increase the DS degree of wettability. For this purpose, Buonocore MG^26^ proposed the pre-conditioning of the tooth surface before the DS placement to improve its adhesion.

The two materials most commonly used are resin-based sealants and glass ionomer cement.^4,5^ The American Dental Association recommends the use of resin sealants as a more effective alternative for sealing pits and fissures because of their higher retentive properties compared to glass ionomer sealants.^27^ However, the latter can be considered an excellent alternative when adequate isolation of the tooth for sealant placement is unfeasible.^27^

The FDI World Dental Federation defined minimal intervention approach as a preventive philosophy, focusing on early detection of carious lesions and efforts to remineralize non-cavitated lesions, providing immediate preventive care to minimize surgical interventions and loss of tooth structure.^28^ The application of pit and fissure sealants in patients with high caries risk is one of the strategies recommended by the Minimal Intervention Dentistry approach. With the advent of this philosophy, there was an increase in the use of DS, as this material helps in the prevention and remineralization of initial dental caries lesions, preserving as much of the dental structure as possible.^24^ Thus, the consolidation of the Minimal Intervention Dentistry precepts may also explain the increase in the number of publications with DS theme from the year 2000 onwards.

The most prominent author in this review was Buonocore MG, with the highest number of publications and citations. Buonocore was an essential and notable researcher in the scientific development of DS. A literature review published in 1996 entitled “Michael Buonocore and the Eastman Dental Center: a historical perspective on sealants” demonstrates the great contributions of this author to this theme.^29^ Buonocore was responsible for developing innovative research in the preparation of the enamel surface with a weak acid to increase the adhesion of an organic plastic chemical sealant and polymerization of a sealant with ultraviolet light. He was also pioneer in relation to minimizing the removal of enamel and dentin in the preparations.^29^ In addition, it has been demonstrated that dental sealants are economical and have excellent retention rates.^29^ This information is in line with the data of the top 100 article, where Buonocore mainly conducted clinical studies on the effectiveness and retention of this material. Other prominent authors in this review were Mayer-Lueckel and Paris S, mostly addressing laboratory studies on DS efficacy and retention. With many citations, Gooch BF and Griffin SO developed five studies together, mainly literature reviews on the efficacy and retention of DS.

The Journal of the American Dental Association and Community Dentistry and Oral Epidemiology journals were those with the highest number of articles in the 100 most cited list. The Journal of the American Dental Association was founded in 1913 to promote research through clinical information.^30^ Community Dentistry and Oral Epidemiology is one of the leading international journals in the field of epidemiological dentistry.^31^ Both journals comprise studies of high relevance in DS and are therefore understood and used as references to assist clinicians make clinical decisions and researchers interpret data and conduct research.^30,31^

The study designs with the greatest predominance among the most cited articles were intervention studies and laboratory studies, which mainly addressed the theme of DS retention and effectiveness in paralyzing carious lesions. These studies mainly address the performance of sealed teeth in dental caries arrest and prevention compared with unsealed teeth.^32,33^ Unlike other bibliometric analyses published in the literature,^15,34^ this bibliometric review focused on intervention studies. As dental caries is considered one of the most prevalent diseases in dentistry, it was probably easier to obtain samples for this type of study.^1^ The term retention was the most frequent theme and the most used keyword by the 100 most cited studies on dental sealants.

The second most addressed theme in this study was the release of bisphenol A (BPA). DS consists mainly of BPA-containing monomers.^35^BPA was recognized in the 1930s as an endocrine-disrupting chemical that alters hormonal function.^35^ However, Fung EY et al.^36^ showed that BPA released orally from a DS might not be absorbed or be present in undetectable amounts in the systemic circulation. This fact explains the several types of research involving this dental material in other areas and the fact that the most addressed theme was DS retention and effectiveness against dental caries. Still, the BPA exposure theme was associated with the top 100 DS studies published in dental research journals and dental materials.

North America was the most prevalent continent in this bibliometric analysis, as the USA was the country with the highest number of articles among the 100 most cited. This is explained by the high level of government investment in American universities and research centers.^13^ Among these centers, the Center for Disease Control and Prevention stands out as one of the institutions with the most articles among the most cited. Such finding is associated with prominent authors from this center (Griffin SO and Gooch BF). The University of Rochester, also located in the USA, was also important in this study and the institution to which Buonocore MG (most frequent author) is affiliated.

One of the strengths of this study is the detailed analysis of the main characteristics of the most frequently cited articles on dental sealants without restricting by year of publication or language. With this, the countries, continents, and institutions that most intensely investigate this topic were identified, providing an outlook on future global scientific progress in this field. The exclusive use of the WoS-CC database can be cited as a limitation of this study, as there are other bibliometric databases such as Scopus and Google Scholar. Based on other important bibliometric analyses in the field of dentistry,^13,14^ it was decided to use only the WoS-CC since this database is widely recommended and used for this type of study.^37^

## Conclusion

In conclusion, the 100 most cited articles on DS were published mainly by North American and European authors, indicating little collaboration with other continents. Buonocore MG, Meyer-Lueckel H, and Paris S were the authors with the highest number of articles. In addition, there was a predominance of interventional and laboratory studies, addressing the retention of DS and its effectiveness in arresting initial dental caries lesions.
